# Metabonomics Study of the Hematopoietic Effect of Medicinal Wine Maoji Jiu on a Blood Deficiency Rat Model by Ultra-High-Performance Liquid Chromatography Coupled to Quadrupole Time-of-Flight Mass Spectrometry and a Pattern Recognition Approach

**DOI:** 10.3390/molecules27123791

**Published:** 2022-06-13

**Authors:** Fanqiang Zeng, Yongli Xu, Yilian Li, Zhigang Yan, Li Li

**Affiliations:** 1 Department of Pharmacy, Guigang City People’s Hospital, The Eighth Affiliated Hospital of Guangxi Medical University, Guigang 537100, China; zeng_fanqiang@126.com (F.Z.); gyyxb2530@163.com (Y.L.); 2 Guangxi Botanical Garden of Medicinal Plants, Nanning 530023, China; xylyzg7898@126.com; 3 Guangxi Institute of Chinese Medicine and Pharmaceutical Science, Nanning 530022, China

**Keywords:** Maoji Jiu, blood deficiency, hematopoietic effect, metabolomics, biomarkers

## Abstract

Maoji Jiu (MJ) is a kind of medicinal wine that has been widely used by Chinese people for many years to nourish and promote blood circulation. The purpose of this study was to investigate the hematopoietic effect of MJ on the metabolism of blood deficient rats and to explore the underlying hematopoietic regulation mechanisms. Blood deficiency model rats were induced by subcutaneous injection of N-acetylphenylhydrazine (APH) and intraperitoneal injection of cyclophosphamide (CTX). The plasma metabolic fingerprints of blood deficiency model rats with and without MJ treatment were obtained by using metabonomics based on ultra-high-performance liquid chromatography coupled to quadrupole time-of-flight mass spectrometry (UHPLC–QTOF/MS). Orthogonal partial least squares-discriminant analysis (OPLS–DA) was used to evaluate the hematopoietic effect of MJ and identify potential biomarkers in the plasma of blood deficiency model rats. The levels of white blood cells (WBC), red blood cells (RBC) and hemoglobin (HGB) and the activity of antioxidant capacity showed a recovery trend to the control group after MJ treatment, while the dose of 10 mL/kg showed the best effect. In this study, thirteen potential biomarkers were identified, which were mainly related to seven metabolic pathways, including linoleic acid metabolism, d-glutamine and d-glutamate metabolism, alanine, aspartate and glutamate metabolism, tryptophan metabolism, pyrimidine metabolism, porphyrin and chlorophyll metabolism and arginine biosynthesis. Metabolomics was applied frequently to reflect the physiological and metabolic state of organisms comprehensively, indicating that the rapid plasma metabonomics may be a potentially powerful tool to reveal the efficacy and enriching blood mechanism of MJ.

## 1. Introduction

Blood deficiency, one of the common syndromes in traditional Chinese medicine (TCM) clinics, is a pathological state induced by organ dystrophy and blood disorder. It was reported that blood deficiency syndrome was often caused by massive blood loss, blood stasis, insufficient hematopoiesis and deficient spleen and stomach (Li et al., 2015). In a physiological condition, such as menstruation and pregnancy, blood deficiency commonly becomes a threat to women and widely affects their health. It was reported that many TCMs had a powerful therapeutic effect on blood deficiency on account of their particular effects and lower toxicity for the past few years [1,2].

According to a long-term practice which was recorded in the *Pharmacopoeia of the People’s Republic of China* (volume I) [3], Maoji Jiu (MJ), as a medicinal wine widely known in China, has notable quality and efficacy for nourishing blood and regulating menstruation. It consists of *Centropus sinensis* (Hechiyajuan in Chinese), *Angelica sinensis* (Oliv.) Diels. (Danggui in Chinese), *Ligusticum striatum* DC. (Chuanxiong in Chinese), *Angelica dahurica* (Hoffm.) Benth. and Hook.f. ex Franch. and Sav. (Baizhi in Chinese), *Carthamus tinctorius* L. (Honghua in Chinese), *Paeonia lactiflora* Pall. (Chishao in Chinese), *Prunus persica* (L.) Batsch (Taoren in Chinese), *Homalomena occulta* (Lour.) Schott. (Qiannianjian in Chinese), *Poria cocos* (Schw.) Wolf (Fuling in Chinese) and some liquor. Some of these TCMs had beneficial effects on blood deficiency, which have been confirmed in serial studies [4,5,6,7,8]. However, the hematopoietic effect and enriching blood mechanism of MJ remained unclear. Due to the complex components and mechanism of TCMs, an efficient and sensitive technique must be introduced to further explore the enriching blood mechanism of MJ.

Metabonomics, as an emerging field of the biological system, is a new technique developed rapidly following the genome and proteomics. It indicates that the biological, physiological and pathological states are connected with exogenous stimulation [6,9]. The status of the organism can be acknowledged through investigating the change in endogenous small molecule metabolites (such as fatty acids, carbohydrates, amino acids, fats and vitamins, etc.). With the variation in metabolic components or content in response to the stimulation and disturbance in biological system, we can investigate the metabolic pathways of the biological system [10,11]. Metabonomics looks upon the human body as a whole and physiological and pathological processes as a dynamic process, which is consistent with “holism” and the “dynamic” of the TCM theory [12]. Therefore, it has been applied widely to research and to developing TCM, with its unique characteristics and superiorities.

Recently, many metabonomics detection technologies, including gas chromatography–mass spectrometry (GC–MS), nuclear magnetic resonance (NMR) spectroscopy and liquid chromatography–mass spectrometry (LC–MS), have been used in metabonomics studies [13,14,15,16]. All of them have been widely applied to metabonomics studies. Among these technologies, LC–MS has an obvious advantage over the others in the metabonomics analysis of large structured databases. Due to the selectivity, sensitivity and reproducibility, LC–MS has recently gained frequent application in metabonomics studies [17,18].

In this paper, the purpose of our study was to explore the hematopoietic effect of MJ on blood deficient rats and elucidate the enriching blood mechanisms of MJ with a metabonomic approach. The plasmatic metabolites in rats were analyzed by ultra-high-performance liquid chromatography coupled to quadrupole time-of-flight mass spectrometry (UHPLC–QTOF/MS) after MJ treatment. According to the multivariate statistical analysis method and metabonomics database, the potential biomarkers were filtrated and identified, and their relevant metabolic pathways were analyzed and confirmed. This study is aimed at exploring the hematopoietic effect and its underlying mechanism of MJ with an overall perspective.

## 2. Results

### 2.1. Peripheral Blood Routine Analysis

**Figure 1:**
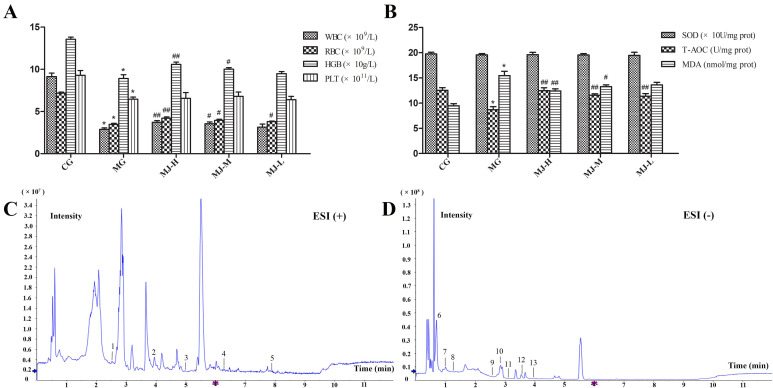
(**A**) Peripheral blood routine analysis. (**B**) Antioxidant activity analysis. (**C**,**D**) Representative base peak intensity chromatogram of plasma samples in MJ-H group derived from UHPLC–QTOF/MS, peak 1, Uracil; 2, Cytidine; 3, Picolinic acid; 4, l-Glutamate; 5, Glutathione disulfide; 6, Linoleic acid; 7, Formylanthranilic acid; 8, Bilirubin; 9, Uridine; 10, Protoporphyrin; 11, 5-Hydroxyindoleacetate; 12, Orotate; 13, l-Tryptophan. (* *p* < 0.01, compared with control group; compared with model group, ^#^ *p* < 0.05, ^##^ *p* < 0.01.)

Blood routine examinations of all the blood samples in tubes were tested using a full-automatic blood cell analyzer, and the results are presented in Figure 1A.

Compared with the control group (CG), the white blood cell (WBC), red blood cell (RBC), hemoglobin (HGB) and platelet (PLT) levels in the model group (MG) presented a significant decreasing tendency, indicating that the blood deficiency model rats were copied successfully. Compared with the model group, the WBC, RBC and HGB levels in the rats of the high dose group of MJ (MJ-H) and middle dose group of MJ (MJ-M) groups showed a meaningful increasing tendency, indicating that MJ can improve the blood routine of the blood deficiency model rats.

### 2.2. Antioxidant Activity Analysis

Liver injury could directly lead to oxidative stress and lipid peroxidation. In this study, we hypothesized that MJ could mitigate liver injury by alleviating oxidative stress and lipid peroxidation. Therefore, three important markers, containing superoxide dismutase (SOD), total antioxidant activity (T-AOC) and malondialdehyde (MDA), were measured to evaluate the antioxidation of MJ. The results showed that N-acetylphenylhydrazine (APH) and cyclophosphamide (CTX) treatment contributed to a significant reduction in the T-AOC activity of liver homogenate. Meanwhile, the MDA levels of liver homogenate increased significantly. Impressively, the two variations induced by APH and CTX were reversed by MJ treatment, displaying in increased T-AOC activity and decreased MDA level (Figure 1B).

As a whole, MJ can improve the antioxidant capacity, protecting the liver injury of the blood deficiency model rats induced by APH and CTX.

### 2.3. The Stability of UHPLC–QTOF/MS System

The quality control (QC) sample was used to examine the stability of the UHPLC–QTOF/MS system. In the whole sequence, the QC sample was run at every six samples in positive and negative modes. The relative standard deviations (RSD) of the retention times and peak areas of all peaks were calculated and the RSD < 30% screening rates of features in positive and negative modes were 96.40% and 96.26%, respectively. The results suggested that the stability of the UHPLC–QTOF/MS system in the whole experiment were high and acceptable.

### 2.4. Metabolic Profiling of Plasma

The representative total ionic chromatograms of the plasma samples were obtained in positive and negative modes by using the UHPLC–QTOF/MS conditions described above. The results showed that the metabolites of the plasma samples could be separated well in the short time of 12 min (Figure 1C,D).

The three crucial data points, including the retention time, peak intensity and exact mass, were imported into SIMCA 14.1 software (Umea, Sweden) for multiple statistical analysis, containing principal component analysis (PCA) and orthogonal partial least-squares discriminant analysis (OPLS–DA).

### 2.5. Multivariate Data Analysis

**Figure 2:**
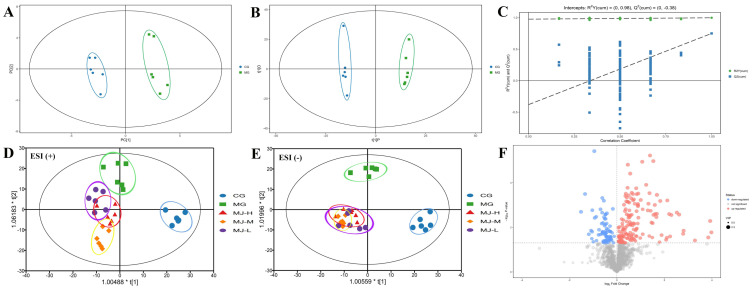
Multivariate data analysis between control and model groups in positive mode. (**A**) PCA score plot. (**B**) OPLS–DA score plot. (**C**) Permutation test of OPLS–DA model. (**D**,**E**) OPLS–DA score plot between the treatment groups and control group. (**F**) Volcano plot.

The data of plasma samples were analyzed by PCA. The PCA score plots presented a very good separation in the plasma samples between the control group and the model group (Figure 2A), indicating that the metabolic profiles of the two groups had changed as a result of APH and CTX treatment. However, the detailed differences in each cluster remained unknown. Therefore, the OPLS–DA was used to better illuminate the different patterns. The results also presented a very good separation in the OPLS–DA score plots between the control group and the model group (Figure 2B).

Seven-fold cross-validation was used to evaluate the predictive ability of the established OPLS-DAOPLS–DA, in which the parameters R^2^Y and Q^2^ are commonly used for evaluating the OPLS–DA model. The value of parameter R^2^ was gradually close to 1, indicating that the building model coincided with the number of samples. At the same time, the value of parameter Q^2^ was also close to 1.0, indicating that the same distribution would be obtained if new samples were added in the model (Figure 2C). Simultaneously, along with permutation retention decreasing and the proportion of Y variation increasing, the parameters R^2^ and Q^2^ of the stochastic model showed a decreasing tendency, indicating that there was no over-fitting phenomenon in the original model. In summary, the results demonstrated that the established OPLS–DA model demonstrated good fitness and predictive ability.

The relative distances between the control group and the others (model group and MJ treatment groups) from the OPLS–DA scores plot of the plasma samples were calculated for quantitative analysis (Figure 2D,E). The average value of the metabolic pattern in the control group was used as the referenced point for the metabolic pattern in the others. In this study, the relative distances between the control group and the others in the OPLS–DA score plots were calculated for evaluating the metabolic differences (Table 1).

Our results showed that the relative distances of the MJ treatment groups in positive and negative modes were closer to the control group in comparison with the model group, indicating that MJ treatment could reverse the abnormal metabolism of the blood deficiency model rats induced by APH and CTX to normal status.

### 2.6. Identification and Quantization of Potential Biomarkers

According to the OPLS–DA model, a loading plot was constructed which presented the contribution of important variables to the difference between two groups. Potential biomarkers were screened on the basis of the variable importance in the projection (VIP) value and significant test from the OPLS–DA model between the control group and the model group. The variables, only under the condition of VIP >1.0 and *p* < 0.05 (*t*-test), were considered to have a meaningful contribution to the OPLS–DA model (Figure 2F). Following the threshold described above, thirteen metabolites were considered as potential biomarkers in the blood deficiency model rats, including five metabolites in positive mode and eight metabolites in negative mode, which are identified and listed in Table 2.

In comparison with the ionic intensity of potential biomarkers between the control group and the model group, nine metabolites, containing uracil, cytidine, l-glutamate, l-arginine, linoleic acid, bilirubin, uridine, protoporphyrin and orotate, were up-regulated, and four metabolites, containing picolinic acid, glutathione disulfide, formylanthranilic acid, 5-hydroxyindoleacetate and l-tryptophan, were down-regulated (Table 2). Intriguingly, the MJ treatment groups showed the tendency to rectify the contents of these abnormal metabolites in connection with a positive dosage relation.

### 2.7. Biological Pathway Analysis

**Figure 3:**
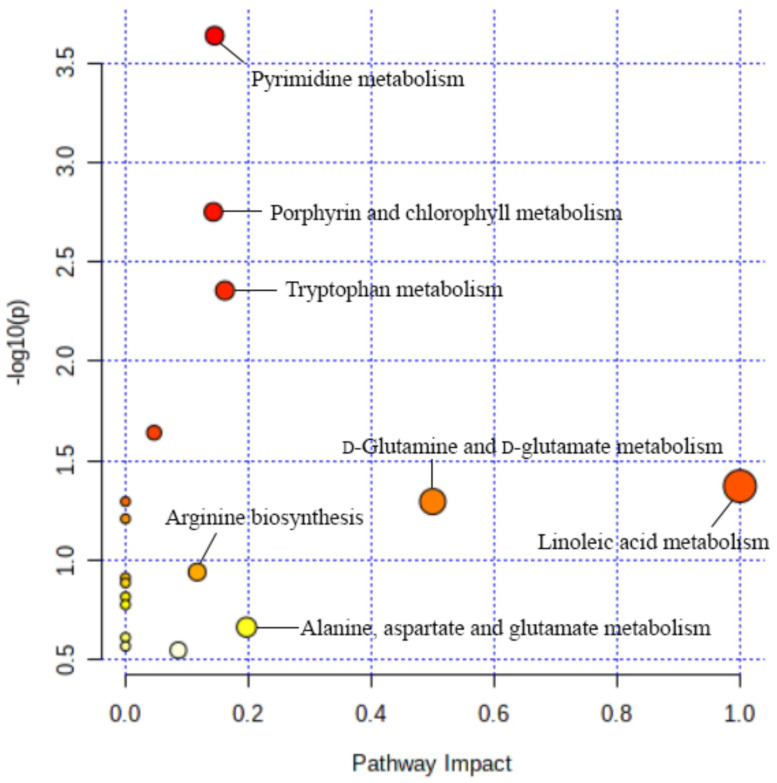
Metabolic pathways of plasma samples of blood deficiency model rats.

The potential biomarkers, listed in Table 2, were imported into MetaboAnalyst 5.0 (https://www.metaboanalyst.ca/, accessed on 14 January 2022) for biological pathway analysis. Seventeen metabolic pathways were established (Figure 3), which were important for the blood deficiency model rats’ response to MJ treatment. Among the seventeen pathways, the impact-values of linoleic acid metabolism, d-glutamine and d-glutamate metabolism, alanine, aspartate and glutamate metabolism, tryptophan metabolism, pyrimidine metabolism, porphyrin and chlorophyll metabolism and arginine biosynthesis were 1.00, 0.50, 0.20, 0.16, 0.15, 0.14 and 0.12, respectively. The metabolic pathways mentioned above were selected as the most important metabolic pathways because the pathway with an impact threshold higher than 0.10 was considered as a potential target pathway [11].

Among the thirteen potential biomarkers identified in the plasma samples of the blood deficiency model rats in this paper, linoleic acid mainly participated in linoleic acid metabolism; l-glutamate was d-glutamine and d-glutamate metabolism, alanine, aspartate and glutamate metabolism, arginine biosynthesis and porphyrin and chlorophyll metabolism; formylanthranilic acid, 5-hydroxyindoleacetate and l-tryptophan were tryptophan metabolism; uracil, cytidine, uridine and orotate were pyrimidine metabolism; bilirubin and protoporphyrin were porphyrin and chlorophyll metabolism.

## 3. Discussion

In modern medicine, the reduction in hemoglobin is commonly considered as blood deficiency, including aplastic anemia, iron deficiency anemia and blood loss anemia [19]. However, blood loss anemia is more similar to the condition of postoperative and postpartum women, with uterine bleeding or chronic bleeding [20]. It was reported that the blood deficiency model induced by APH and CTX in this study was more in accordance with the internal environment of blood deficiency [6]. APH has slowly progressive and oxidative damage on RBC because of its strong oxidation, leading to hemolytic anemia of the body [21]. CTX can decrease peripheral blood cells and hematopoietic stem cells in the marrow, resulting in anemia and immune deficiency [22]. Furthermore, it was reported that APH and CTX would destroy the antioxidant system, resulting in weak antioxidant capacity of the body [23]. In our study, the results demonstrated that MJ could increase the levels of WBC, RBC and HGB and the activity of T-AOC and decrease the contents of MDA in the blood deficiency model rats.

**Figure 4:**
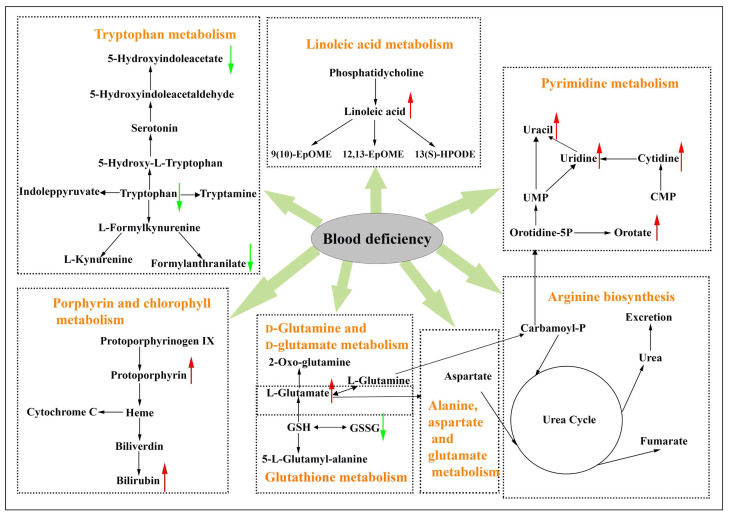
Correlation networks of main potential biomarkers related to blood deficiency and the effects of treatment for blood deficiency. The contents of potential biomarkers in model group compared to control group were marked with (↑) up-regulated and (↓) down-regulated.

In this paper, metabolomics was used for exploring the enriching blood mechanism of MJ, and thirteen potential biomarkers, such as linoleic acid, l-glutamate, l-tryptophan and uracil, etc., were filtrated and identified in the plasma samples combined with PCA and OPLS–DA. Among these metabolites, linoleic acid, belonging to n-6 fatty acid, is an important component of free fatty acids, and its metabolism belongs to linoleic acid metabolism (Figure 4). The existence of unsaturated double bonds in the structure of linoleic acid is easily oxidized by free radical attack, resulting in large amounts of free radicals and active substances [24,25]. Large amounts of reactive oxygen species are produced, exceeding the scavenging capacity of the body, which would release to outside of the cell and attack surrounding cells and tissues, resulting in damage [26]. The concentrations of linoleic acid in the model group obviously increased compared to the control group, which was in accordance with the increased concentrations of linoleic acid in the animals with blood deficiency, including rats and mice [22,27]. Down-regulation of linoleic acid in the MJ treatment groups indicates that MJ could recover the dysfunction of linoleic acid metabolism and reduce injury to cells and tissue in the blood deficiency model rats.

l-Glutamate, one of the most abundant amino acids in body, is related to d-glutamine and d-glutamate metabolism, arginine biosynthesis and alanine, aspartate and glutamate metabolism. The concentration of l-glutamate in the synaptic cleft is significantly higher than in blood plasma under normal physiological conditions. However, the concentration of l-glutamate in blood plasma increases substantially in numerous pathological conditions [28]. Glutathione (GSH) is an endogenous three amino acid peptide in cells and serves as the main antioxidant in the biological system. GSH mainly translates into l-glutamate, glutathione disulfide (GSSG) and 5-l-glutamyl-alanine in the body, and all of them take part in glutathione metabolism. In this paper, down-regulation of GSSG and up-regulation of l-glutamate may be induced by abnormal transformation of GSH in the metabolic pathway, which will cause the abnormality of d-glutamine and d-glutamate metabolism, glutathione metabolism, alanine, arginine biosynthesis, and aspartate and glutamate metabolism directly (Figure 4). It was reported that high concentrations of l-glutamate play a double role in initiating and controlling the process of nerve cell death in the process of excitatory toxic injury of neurons [29]. Increased levels of l-glutamate and decreased levels of GSSG were observed in the MJ treatment groups, indicating that the therapeutic effects of MJ may be based on the regulation of the dysfunction of all metabolic pathways mentioned above to protect the body against damage.

Tryptophan, as the precursor of several active compounds containing kynurenine and serotonin (5-hydroxytryptamine), is crucial for numerous important metabolic functions [30]. There are four different pathways of tryptophan metabolism in mammalian systems (Figure 4). The serotonergic pathway is the most widely known, being active in platelets and neurons, and it translates tryptophan into 5-hydroxytryptophan and then into serotonin. The kynurenine pathway is an alternate route of tryptophan metabolism, which translates tryptophan into nicotinamide adenine dinucleotide and its phosphorylated form through several intermediates [31]. Abnormal metabolism of kynurenine is closely related to many diseases, including aplastic anemia, immune dysfunction and depression [32]. The contents of l-tryptophan decreased significantly in the plasma samples of the blood deficiency model rats, which corresponds with the decreased content of l-tryptophan in the plasma samples of the blood deficiency mice [33]. Simultaneously, the metabolites of 5-hydroxyindoleacetate in the serotonergic pathway and formylanthranilic acid in the kynurenine pathway decreased significantly in the plasma sample of the blood deficiency model rats, indicating that the two pathways of tryptophan metabolism were affected in the blood deficiency model rats. The levels of l-tryptophan, 5-hydroxyindoleacetate and formylanthranilic acid increased in the MJ treatment groups compared to the model group, indicating MJ treatment may improve the biosynthesis of tryptophan to increase tryptophan metabolism.

Protoporphyrin and bilirubin are two metabolites that take part in porphyrin and chlorophyll metabolism. Protoporphyrin is a heterocyclic organic compound composed of four pyrrole rings, which is the final intermediate in the heme biosynthetic pathway (Figure 4) [34]. Heme is an essential component of hemoglobin as well as a variety of physiologically important hemoproteins [35]. The contents of protoporphyrin increased significantly in the plasma samples of the blood deficiency model rats, which corresponded with the increased content of protoporphyrin in the plasma samples of the blood deficiency model rats induced by APH [36]. Up-regulation of protoporphyrin in the model group may be related to the blocked heme synthesis. Bilirubin mainly comes from the metabolism of heme in red blood cells. Hemolytic anemia of the body can be induced by APH because it has a slowly progressive and oxidative damage on RBC [21]. The contents of bilirubin increased significantly in the plasma samples of the blood deficiency model rats, which might be associated with the increased heme metabolism in the damaged RBC induced by APH. The increased contents of protoporphyrin and bilirubin were observed in the MJ treatment groups, indicating that MJ treatment could recover the dysfunction of porphyrin and chlorophyll metabolism in the blood deficiency model rats induced by APH and CTX.

The metabolites, including oratate, cytidine, uridine and uracil, participate in pyrimidine metabolism (Figure 4) and belong to nucleotide metabolism, containing the pyrimidine degradation, pyrimidine ribonucleotide biosynthesis, uridine monophosphate biosynthesis and pyrimidine deoxyribonuleotide biosynthesis. Previous studies showed that the metabolic disorders of pyrimidine nucleotide can present as anemia, resulting in the accumulation of excess pyrimidine nucleotides [37,38]. In our study, the blood deficiency model rats induced by APH and CTX showed a significant increasing tendency of the metabolites, including oratate, cytidine, uridine and uracil, which might be associated with the increased pyrimidine metabolism or the decreased pyrimidine degradation. The contents of oratate, cytidine, uridine and uracil decreased in the MJ treatment groups, which indicated that the protective effect of MJ might be associated with the regulation of pyrimidine metabolism or pyrimidine degradation.

## 4. Materials and Methods

### 4.1. Preparation of MJ

Hechiyajuan was obtained from Pingnan (Guangxi, China). Danggui, Chuangxiong, Baizhi, Honghua, Chishao, Taoren, Qiannianjian and Fuling were purchased from Yancheng Buyi Pharmacy Co. Ltd. (Jiangsu, China). In this study, the preparation of MJ was performed in a traditional and classical way which was recorded in the *Pharmacopoeia of the People’s Republic of China* (volume I) [3]. The dried Hechiyajuan (80 g, the viscera and plumages were removed) was steamed with a boiler for 15 min and then placed in a glass bottle with 40% of appropriate liquor (4.20 L). Then, the bottle was sealed for 25 days. The remaining medicinal materials, including Danggui (80 g), Chuanxiong (80 g), Baizhi (80 g), Honghua (80 g), Chishao (7.5 g), Taoren (7.5 g), Qiannianjian (80 g), Fuling (10 g) and the remaining liquor (the total volume from the last time and from this time was 8.40 L) were added into the bottle. The bottle was sealed and placed again for 55 days. The filtrate was obtained and evaporated with a rotary evaporator under vacuum at 50 °C. The content of ethanol in the concentrated solution was adjusted to 40%, and then the MJ sample (a total volume of 4.20 L) was obtained.

### 4.2. Blood Deficiency Model Building and Administration

Thirty female Sprague-Dawley rats (180~220 g) were provided by the Experimental Animal Center of Guangxi Medical University (Nanning, China). The animal experiment was approved by the Ethics Committee of the Guangxi Botanical Garden of Medicinal Plants (protocol code 20170301). All the rats were randomly divided into five groups of six rats per group: control group, model group, high-dose group of MJ (MJ-H), medium-dose group of MJ (MJ-M) and low-dose group of MJ (MJ-L). Blood deficiency model construction mainly referred to Li’s method [6]. Except for the control group, all the rats were subcutaneously injected with 2% APH (Shanghai, China) normal saline at doses of 20 mg/kg and 10 mg/kg on day 1 and 4, respectively. On day 4, 2 h after subcutaneous injection of 2% APH normal saline, the rats were intraperitoneally injected with 15 mg/kg CTX (Baxter Oncology GmbH, Germany) normal saline for four consecutive days. The rats in the MJ-H, MJ-M and MJ-L groups intragastrically received a 10 mL/kg, 5 mL/kg and 2.5 mL/kg MJ sample, respectively, twice a day for 10 consecutive days from day 1. At the same time, the rats in the control group and model group received the same amount of normal saline.

### 4.3. Sample Preparation

At the end of treatment, peripheral blood and abdominal aorta blood samples in all rats were collected for routine blood tests and UHPLC–QTOF/MS analysis, respectively. The aorta blood samples were immediately centrifuged in a condition of 3000× *g* rpm and 4 °C for 10 min. Then, the plasma samples were obtained and stored at −80 °C. The livers of all the rats were obtained for the antioxidant enzyme and lipid peroxidation estimations by using commercially available kits.

The plasma samples were thawed at room temperature before pre-treatment. The extraction liquid (350 μL), including methanol, methanol and water and their volume ratio in sequence, was 2:2:1 and was added into each plasma sample (100 μL) for precipitating protein. As an internal standard, L-2-chlorophenylalanineas (20 μL, 1 mg/mL) was added to each mixture’s sample. Afterwards, the mixture samples were vortexed for 30 s and ultrasonically extracted in an ice water bath for 10 min. Then, the mixtures were incubated at −20 °C for 60 min and centrifuged in a condition of 12,000× *g* rpm and 4 °C for 15 min. Next, the supernatant (400 μL) of each mixture was transferred into a new tube and dried in a vacuum desiccator. The residuums of plasma samples were dissolved in 50% acetonitrile solution (100 μL). The mixtures were vortexed for 30 s, sonicated in an ice water bath for 10 min, and centrifuged in a condition of 12,000× *g* rpm and 4 °C for 15 min. Finally, 60 μL of the supernatant from each mixture was obtained and transferred into a sample bottle for UPLC/MS analysis. Then, the QC sample was taken from all samples and mixed together, which was used to validate the stability of the UPLC/MS system.

### 4.4. UHPLC–QTOF/MS Conditions

An Agilent Technologies 1290 LC (Agilent Technologies, Santa Clara, CA, USA) equipped with BEH Amide column (2.1 mm i.d. × 100 mm, 1.7 μm) was applied to separate the metabolites in the plasma samples. The column was maintained at 35 °C. The mobile phase consists of mobile phase A (25 mmol/L NH_4_OAc and 25 mmol/L NH_4_OH in water, pH = 9.75) and mobile phase B (acetonitrile). The flow rate always stayed at 500 μL/min with a gradient elution (0–1.0 min, 99% A; 1.0–8.0 min, 99–0% A; 8.0–10.0 min, 0% A; 10.0–10.1 min, 0–99% A; 10.1–12.0 min, 99% A) throughout the whole analysis. An aliquot of 2 μL of each plasma sample was injected into the BEH Amide column. MS analysis was carried out on an AB SCIEX^TM^ × 5500 Triple TOF mass spectrometer (AB SCIE^TM^, Foster City, CA, USA) in positive and negative ion modes. The electrospray ionization (ESI) source conditions were set as follows: collision energy was 35 eV, ion source gas 1 was 60 Pa, ion source gas 2 was 60 Pa, curtain gas was 30 Pa, source temperature was 550 °C and ion spray voltage was 5500 V in positive mode and −4500 V in negative mode.

### 4.5. Pattern Recognition Analysis and DATA Processing

All mass data obtained were aligned using SIMCA 14.1 software according to the retention time and the *m*/*z* value of each ion signal. Then, all the ionic signals detected in each plasma sample were normalized to the sum of the total ionic intensity of each chromatogram. Afterwards, the analytical methods in SIMCA 14.1 software, including PCA and OPLS–DA, were used to analyze the ionic signals in positive and negative modes. The OPLS–DA score plot was described by the seven-fold cross-validation parameters R^2^Y and Q^2^, representing the total explanatory variable of the Y-matrix and the predictability of the model, and then the validity of the model was tested by permutation test. Potential biomarkers were chosen on the basis of the parameters of VIP from OPLS–DA, and they were further analyzed according to biochemical databases. The relative distances—the separation of plasma samples between the control group and the other groups in the OPLS–DA score plot—were calculated with the mean value. Finally, the potential biomarkers were imported into MetaboAnalyst 5.0 (http://www.metaboanalyst.ca/, accessed on 14 January 2022) for biological pathway analysis.

### 4.6. Data Analysis

The experimental data were analyzed using SPSS 20.0 software. Statistical significance between the groups was assessed by *t*-test and a one-way analysis of variance. The results were presented as the mean ± SE, and a value of *p* < 0.05 was considered to be statistically significant.

## 5. Conclusions

In this paper, the constructed UPLC/MS-based plasmic metabonomics approach was applied to explore the enriching blood mechanism of MJ on the blood deficiency model rats induced by APH and CTX. Multivariate statistical analysis and MetaboAnalyst analysis were combined to obtain comprehensive metabolomics tracks and pathways of blood deficiency model rats. The hematopoietic effects of MJ were evaluated for regulating the altered pathway. A total of thirteen metabolites related to blood deficiency were tentatively filtrated and identified, and the variation in these metabolites could be reversed by MJ. MetaboAnalyst analysis showed that the hematopoietic effect of MJ might be related to regulating the abnormality of linoleic acid metabolism, d-glutamine and d-glutamate metabolism, alanine, aspartate and glutamate metabolism, tryptophan metabolism, pyrimidine metabolism, porphyrin and chlorophyll metabolism and arginine biosynthesis. This study elucidated the biological explanations of MJ for treating blood deficiency from the perspective of metabolomics, providing a methodological reference for exploring the mechanism of TCM.

## Figures and Tables

**Table 1:** The relative distances between the treatment groups and the control group from the OPLS–DA score plots of the plasma samples.

ESI Mode	Control Group		Model Group	MJ-H	MJ-M	MJ-L
	*x*-Axis	*y*-Axis				
+	23.32	−4.23	32.62 ± 5.03	27.40 ± 2.77 *	32.05 ± 2.53	33.45 ± 2.85
−	23.39	−6.81	34.80 ± 2.69	30.08 ± 3.46 *	32.63 ± 0.98	28.93 ± 4.07 *

* *p* < 0.05, compared with model group.

**Table 2:** Metabolites selected as potential biomarkers characterized in plasma profile and their change trends (n = 6 in each group).

No.	ESI Mode	t_R_ (min)	*m*/*z*	VIP	*p*	HMDB ID	Metabolites	Trend in Model Group ^a^	Trend in MJ-H Group ^b^	Trend in MJ-M Group ^b^	Trend in MJ-L Group ^b^
1	+	2.53	113.0342	2.22	0.0025	00300	Uracil	↑ **	↓ **	↓ **	↓ *
2	+	3.71	266.0743	2.22	0.0011	00089	Cytidine	↑ **	↓ *	↓ *	↓
3	+	5.04	124.0387	1.96	0.0157	02243	Picolinic acid	↓ **	↑ *	↑	↑
4	+	6.21	148.0602	1.40	0.0489	00148	L-Glutamate	↑ *	↓ *	↓	↓
5	+	7.85	613.5462	2.04	0.0003	03337	Glutathione disulfide	↓ **	↑ *	↑	↑
6	−	0.75	279.2280	1.79	0.0066	00673	Linoleic acid	↑ **	↓ *	↓ *	↓
7	−	1.00	164.0327	1.49	0.0093	04089	Formylanthranilic acid	↓ **	↑ **	↑ *	↑
8	−	1.27	583.2440	1.20	0.0307	00054	Bilirubin	↑ **	↓ *	↓ *	↓
9	−	2.57	265.0383	1.56	0.0197	00296	Uridine	↑ **	↓ *	↓	↓
10	−	2.84	561.2397	1.93	0.0013	00241	Protoporphyrin	↑ **	↓ **	↓ *	↓ *
11	−	3.13	190.0476	1.67	0.0022	00763	5-Hydroxyindoleacetate	↓ **	↑ **	↑ **	↑ *
12	−	3.62	155.0065	1.34	0.0368	00226	Orotate	↑ **	↓ **	↓ *	↓
13	−	3.98	203.0791	1.66	0.0036	00929	L-Tryptophan	↓ **	↑ *	↑	↑

^a^ Change trend compared with control group. ^b^ Change trend compared with model group. The levels of potential biomarkers were labeled with (↓) down-regulated and (↑) up-regulated (* *p* < 0.05; ** *p* < 0.01).

## Data Availability

All processed data for the study are included within the manuscript. Raw datasets are available from the corresponding author on reasonable request.

## Sample Availability

Samples of the compounds are not available from the authors.

## References

[1] Marsh J.C., Bacigalupo A., Schrezenmeier H., Tichelli A., Risitano A.M., Passweg J.R., Killick S.B., Warren A.J., Foukaneli T., Aljurf M. (2012). Prospective study of rabbit antithymocyte globulin and cyclosporine for aplastic anemia from the EBMT Severe Aplastic Anaemia Working Party. Blood.

[2] Scheinberg P., Wu C.O., Nunez O., Scheinberg P., Boss C., Sloand E.M., Young N.S. (2009). Treatment of severe aplastic anemia with a combination of horse antithymocyte globulin and cyclosporine, with or without sirolimus: A prospective randomized study. Haematologica.

[3] Editorial Committee of Chinese Pharmacopoeia (1977). Pharmacopoeia of the People’s Republic of China.

[4] Li S., Lin H., Qu C., Tang Y., Shen J., Li W., Yue S., Kai J., Shang G., Zhu Z. (2015). Urine and plasma metabonomics coupled with UHPLC-QTOF/MS and multivariate data analysis on potential biomarkers in anemia and hematinic effects of herb pair Gui-Hong. J. Ethnopharmacol..

[5] Li W., Tang Y., Guo J., Huang M., Li W., Qian D., Duan J. (2011). Enriching blood effect comparison in three kinds of blood deficiency model after oral administration of drug pair of Angelicae Sinensis Radix and Chuanxiong Rhizoma and each single herb. Chin. J. Chin. Mater. Med..

[6] Li W., Tang Y., Guo J., Shang E., Qian Y., Wang L., Zhang L., Liu P., Su S., Qian D. (2014). Comparative metabolomics analysis on hematopoietic functions of herb pair Gui-Xiong by ultra-high-performance liquid chromatography coupled to quadrupole time-of-flight mass spectrometry and pattern recognition approach. J. Chromatogr. A.

[7] Zhang J.J., Huang Y.F., Wang L.L., Li W., Wang J.X., Wang C., Qu S.S. (2013). Comparative study on effects of blood enriching on mouse model of blood deficiency syndrome induced by compound method of bleeding, starved feeding and exhausting of Paeoniae Radix Alba and Paeoniae Radix Rubra, paeoniflorin and albiflorin. Chin. J. Chin. Mater. Med..

[8] Zeng F., Xu Y., Jiang S., Zhang Y., Zhao C., Li L. (2016). Study on blood enriching effect of Centropus sinensis dried body alcohol extract on blood-deficiency model mice. China Pharm..

[9] Nicholson J.K., Lindon J.C., Holmes E. (1999). ‘Metabonomics’: Understanding the metabolic responses of living systems to pathophysiological stimuli via multivariate statistical analysis of biological NMR spectroscopic data. Xenobiotica.

[10] Lao Y.M., Jiang J.G., Yan L. (2009). Application of metabonomic analytical techniques in the modernization and toxicology research of traditional Chinese medicine. Br. J. Pharmacol..

[11] Wang X., Yang B., Sun H., Zhang A. (2012). Pattern recognition approaches and computational systems tools for ultra performance liquid chromatography-mass spectrometry-based comprehensive metabolomic profiling and pathways analysis of biological data sets. Anal. Chem..

[12] Jiang M., Lu C., Zhang C., Yang J., Tan Y., Lu A., Chan K. (2012). Syndrome differentiation in modern research of traditional Chinese medicine. J. Ethnopharmacol..

[13] Ebrahimi F., Ibrahim B., Teh C.H., Murugaiyah V., Chan K.L. (2016). Urinary NMR-based metabolomic analysis of rats possessing variable sperm count following orally administered Eurycoma longifolia extracts of different quassinoid levels. J. Ethnopharmacol..

[14] Li S., Lin H., Tang Y., Li W., Shen J., Kai J., Yue S., Shang G., Zhu Z., Shang E. (2015). Comparative metabolomics analysis on invigorating blood circulation for herb pair Gui-Hong by ultra-high-performance liquid chromatography coupled to quadrupole time-of-flight mass spectrometry and pattern recognition approach. J. Pharm. Biomed. Anal..

[15] Liu M., Liu X., Wang H., Xiao H., Jing F., Tang L., Li D., Zhang Y., Wu H., Yang H. (2016). Metabolomics study on the effects of Buchang Naoxintong capsules for treating cerebral ischemia in rats using UPLC-Q/TOF-MS. J. Ethnopharmacol..

[16] Su Z.H., Li S.Q., Zou G.A., Yu C.Y., Sun Y.G., Zhang H.W., Gu Y., Zou Z.M. (2011). Urinary metabonomics study of anti-depressive effect of Chaihu-Shu-Gan-San on an experimental model of depression induced by chronic variable stress in rats. J. Pharm. Biomed. Anal..

[17] Gu Q., David F., Lynen F., Rumpel K., Dugardeyn J., Van Der Straeten D., Xu G., Sandra P. (2011). Evaluation of automated sample preparation, retention time locked gas chromatography-mass spectrometry and data analysis methods for the metabolomic study of Arabidopsis species. J. Chromatogr. A.

[18] Hong Z., Lin Z., Liu Y., Tan G., Lou Z., Zhu Z., Chai Y., Fan G., Zhang J., Zhang L. (2012). Innovative microwave-assisted oximation and silylation procedures for metabolomic analysis of plasma samples using gas chromatography-mass spectrometry. J. Chromatogr. A.

[19] Trost L.B., Bergfeld W.F., Calogeras E. (2006). The diagnosis and treatment of iron deficiency and its potential relationship to hair loss. J. Am. Acad. Dermatol..

[20] Shi X., Tang Y., Zhu H., Li W., Li Z., Li W., Duan J.A. (2014). Comparative tissue distribution profiles of five major bio-active components in normal and blood deficiency rats after oral administration of Danggui Buxue Decoction by UPLC-TQ/MS. J. Pharm. Biomed. Anal..

[21] Barreda D.R., Hanington P.C., Belosevic M. (2004). Regulation of myeloid development and function by colony stimulating factors. Dev. Com. Immunol..

[22] Ji P., Wei Y., Hua Y., Zhang X., Yao W., Ma Q., Yuan Z., Wen Y., Yang C. (2018). A novel approach using metabolomics coupled with hematological and biochemical parameters to explain the enriching-blood effect and mechanism of unprocessed Angelica sinensis and its 4 kinds of processed products. J. Ethnopharmacol..

[23] Jiang Y., Di Z., Wang Y., An M., Hu J., Jin Y., Zhang Z., Zhang Y. (2019). Study on anti-fatigue, anti-oxidative and hemostatic effects of small molecule Asini Corii Colla. Chin. Pharmacol. Bull..

[24] Kaikkonen J.E., Kresanov P., Ahotupa M., Jula A., Mikkilä V., Viikari J.S., Kähönen M., Lehtimäki T., Raitakari O.T. (2014). High serum n6 fatty acid proportion is associated with lowered LDL oxidation and inflammation: The Cardiovascular Risk in Young Finns Study. Free Radic. Res..

[25] Nowak J.Z. (2013). Oxidative stress, polyunsaturated fatty acids-derived oxidation products and bisretinoids as potential inducers of CNS diseases: Focus on age-related macular degeneration. Pharmacol. Rep..

[26] Zhang M., Wang A., He W., He P., Xu B., Xia T., Chen X., Yang K. (2007). Effects of fluoride on the expression of NCAM, oxidative stress, and apoptosis in primary cultured hippocampal neurons. Toxicology.

[27] Yuan T., Fan W.B., Cong Y., Xu H.D., Li C.J., Meng J., Bao N.R., Zhao J.N. (2015). Linoleic acid induces red blood cells and hemoglobin damage via oxidative mechanism. Int. J. Clin. Exp. Pathol..

[28] Ganor Y., Levite M. (2014). The neurotransmitter glutamate and human T cells: Glutamate receptors and glutamate-induced direct and potent effects on normal human T cells, cancerous human leukemia and lymphoma T cells, and autoimmune human T cells. J. Neural. Transm..

[29] Mayor D., Tymianski M. (2018). Neurotransmitters in the mediation of cerebral ischemic injury. Neuropharmacology.

[30] Sibon D., Coman T., Rossignol J., Lamarque M., Kosmider O., Bayard E., Fouquet G., Rignault R., Topçu S., Bonneau P. (2019). Enhanced Renewal of Erythroid Progenitors in Myelodysplastic Anemia by Peripheral Serotonin. Cell Rep..

[31] Dale W.E., Dang Y., Brown O.R. (2000). Tryptophan metabolism through the kynurenine pathway in rat brain and liver slices. Free Radic. Biol. Med..

[32] Tang J., Chen R., Liao L. (2012). Simultaneous determination of kynurenine and tryptophan in human serum by HPLC. China Pharm..

[33] Li W.X., Huang M.Y., Tang Y.P., Guo J.M., Shang E.X., Wang L.Y., Qian D.W., Duan J.A. (2013). Metabolomic study of the action mechanism of nourishing blood effect of fo-shou-san on blood deficiency mice. Acta. Pharm. Sin..

[34] Sachar M., Anderson K.E., Ma X. (2016). Protoporphyrin IX: The Good, the Bad, and the Ugly. J. Pharmacol. Exp. Ther..

[35] Dailey H.A., Meissner P.N. (2013). Erythroid heme biosynthesis and its disorders. Cold Spring Harb. Perspect. Med..

[36] Zhang Y., Fei Q.Q., Wang J., Zhu F.X., Chen Y., Tang D.Q., Chen B. (2019). Study on blood enrichment mechanism of steamed notoginseng based on metabolomics method. Chin. J. Chin. Mater. Med..

[37] Löffler M., Fairbanks L.D., Zameitat E., Marinaki A.M., Simmonds H.A. (2005). Pyrimidine pathways in health and disease. Trends Mol. Med..

[38] Paglia D.E., Valentine W.N. (1975). Characteristics of a pyrimidine-specific 5′-nucleotidase in human erythrocytes. J. Biol. Chem..

